# Transfer-Learning-Based Estimation of the Remaining Useful Life of Heterogeneous Bearing Types Using Low-Frequency Accelerometers

**DOI:** 10.3390/jimaging9020034

**Published:** 2023-02-04

**Authors:** Sebastian Schwendemann, Axel Sikora

**Affiliations:** Institute of Reliable Embedded Systems and Communication Electronics (ivESK), Offenburg University of Applied Sciences, 777652 Offenburg, Germany

**Keywords:** transfer learning, intermediate domain, remaining useful life, predictive maintenance

## Abstract

Deep learning approaches are becoming increasingly important for the estimation of the Remaining Useful Life (RUL) of mechanical elements such as bearings. This paper proposes and evaluates a novel transfer learning-based approach for RUL estimations of different bearing types with small datasets and low sampling rates. The approach is based on an intermediate domain that abstracts features of the bearings based on their fault frequencies. The features are processed by convolutional layers. Finally, the RUL estimation is performed using a Long Short-Term Memory (LSTM) network. The transfer learning relies on a fixed-feature extraction. This novel deep learning approach successfully uses data of a low-frequency range, which is a precondition to use low-cost sensors. It is validated against the IEEE PHM 2012 Data Challenge, where it outperforms the winning approach. The results show its suitability for low-frequency sensor data and for efficient and effective transfer learning between different bearing types.

## 1. Introduction

Bearings are used in many rotational industrial machines, where they are a critical component because, in the case of an unexpected failure, they can lead to a failure of the machine as a whole. If the machine is part of a production line, the entire line can suffer from the resulting downtimes, causing significant cost and production backlogs. Numerous fault diagnosis methods have been developed and applied to counteract this [1,2,3,4]. These diagnosis methods are based on measurements that are recorded and analyzed in conjunction with further information by experts to detect or predict a defect. Statistical methods in general and Artificial Intelligence (AI) in particular have been used to optimize the analysis [5,6]. These methods promise timely diagnosis and predictive maintenance processes to reduce unplanned downtime and thus increase production efficiency and operational reliability [7].

For the case of predictive maintenance, the estimation of the Remaining Useful Life (RUL) of bearings is of particular interest. There are classical machine learning approaches that, for instance, rely on Support Vector Machines (SVMs) [8]. However, these approaches use features of the time domain, e.g., Vogl and Donmez [9], or rely on features of the time–frequency domain, such as Prudhom et al. [10]. Recently, these approaches have been supplemented with deep learning approaches, which use models based on artificial neural networks arranged in ‘deep’ stacked layers, such as Convolutional Neural Networks (CNN) [11] and Long Short-Term Memory (LSTM) networks [12]. In general, the current deep learning approaches are based on the usage of a high-resolution sensor as the input—either using direct time-series sensor data [13] or through transformations such as a Short-Time Fourier Transformation (STFT) [14].

Nevertheless, deep learning approaches can suffer from limited adaptability if trained for one type of dataset (source) and applied to a different type (target). This can be the case for bearings when a trained model is used for a dataset with different operating conditions or physical characteristics of the bearing [15]. In such cases, deep learning models must be recreated from scratch, leading to an inefficient use of computational resources. For instance, one bearing type may be replaced by another in the same machine, or a different workpiece may be produced. This leads to many different applications of bearings, which results in the common problem of the widespread insufficient availability of datasets for a new domain [16]. Furthermore, in many industrial scenarios, collecting and labeling of large datasets are also too laborious to be applicable [17]. In other words, deep learning approaches are potentially afflicted with limited usability in practical industrial applications.

One possible solution to address the above-mentioned issues of deep learning models for predictive maintenance tasks for machines is to use transfer learning. Here, a neural network is trained for a new target domain with only a few samples and by additionally using source domain knowledge. As a result, transfer learning decreases the computation time compared to training a neural network from scratch [18]. Today, transfer learning approaches are widely used in many applications, such as speech recognition, pattern recognition, and image classification [7]. In addition, they are also widely used for bearing classification and RUL tasks between different process conditions or bearing types [19,20]. Approaches for the RUL estimation are often based on LSTMs because of their ability to analyze historical time-series data to predict the future [18]. This is also evident in their successful usage in the RUL estimation of bearings [13,21].

Most of the current transfer learning approaches for the RUL estimation are between different process conditions. A recent work is, for instance, the one of Cheng et al. [22]. They use a CNN to estimate the RUL of bearings of the 2012 IEEE PHM Data Challenge dataset, which contains only one type of bearings. The transfer task in their setup is to transfer knowledge of the fault behavior from one bearing to another. Therefore, they split all available datasets into groups of different fault behaviors. The unsupervised transfer learning is achieved using the Multi Kernel Maximum Mean Discrepancy (MK-MMD) as an additional loss function. The MK-MMD uses the input of the target and the source domain and adjusts the difference between them by learning domain invariant features. The authors compare the accuracy of an approach with transfer learning versus one without it and find the transfer learning approach to obtain higher accuracy.

In contrast to the previously mentioned transfer learning approaches between different operating conditions, only a few solutions exist for the transfer learning task between different bearing types. To the best of the authors’ knowledge, only two research works are available today. Both are explained in the following.

The work of Xia et al. [21] uses, like most of the other RUL approaches, the dataset of the 2012 IEEE PHM Data Challenge as the target domain dataset, where the source domain dataset is that of Case Western Reserve University [23]. This approach is based on the raw sensor signal as the input and has two parts: A Fault Knowledge Transfer Neural Network (FTNN) and a convolutional LSTM ensemble network. The FTNN has three convolutional layers, each followed by a pooling layer. This neural network is first trained with the target domain dataset. The transfer learning starts with the pretrained but not fixed convolutional layers. Then, this network is trained with inputs from the source and target domains simultaneously. Maximum Mean Discrepancy (MMD), a discrepancy-based algorithm to measure the difference between two distributions, is used for this purpose. The output of the trained FTNN is used as the input for the LSTM ensemble network. This network has n parallel LSTM networks with an identical layout. Each of the n networks is for one bearing condition. As the last step, an ensemble mechanism is used to weigh the outputs of the LSTMs to obtain the correct RUL. They use their own root mean square error (RMSE)-based validation setup to validate their approach. The results show an RMSE reduction of up to 48.61% compared to other self-implemented methods.

Huang et al. [13] propose a similar approach for transfer learning between different bearing types. They feed the raw sensor input into convolutional and pooling layers. Their output is used as the input of an LSTM. Its output, in turn, is used as the input for fully connected layers to estimate the RUL. They use their own backpropagation algorithm, called the Adaptive Hybrid High Power Multi-dimensional Gradient algorithm (AHHPMG). This algorithm considers the temporal correlation of the measurement points in the training data. As a first step, they pretrain the network with the source RUL dataset. Afterward, the pretrained network is trained with the target domain data. The target dataset is not split-bearing instance-based, which means that samples of one bearing are in the training and testing datasets. In our opinion, this is not a valid proceeding since this is only a complex linear interpolation of the RUL values of the training samples.

To sum up, there are many transfer learning approaches between different bearing conditions but only a few between different bearing types. The approaches between different bearing types use the 2012 IEEE PHM Data Challenge dataset and its measurements of a high-resolution sensor, which has a frequency range of up to 25,600 Hz. Moreover, both approaches use their own evaluation methods. Therefore, the results are not comparable with most other approaches, which often use the 2012 IEEE PHM Data Challenge setup and the scoring approach of this challenge for the evaluation.

In addition to the low number of samples, industrial applications often face another challenge: they must show a good performance and a good return on investment. This need competes against the usage of accelerometers with high sampling rates of more than 20 kHz that are available [24] but expensive, especially when more than one accelerometer is used. For instance, two sensors are needed to cover all possible fault positions because, depending on the use case, either the inner or outer ring of the bearing is fixed. In addition, bearings are used under radial load. Therefore, a horizontal and a vertical sensor are needed to measure the accelerations of the fixed ring in every possible direction. Triaxial accelerometers can also be used to avoid the need to use two or three separate accelerometers. Instead, they combine three sensors in one housing, making it possible to simultaneously record accelerations in three orthogonal axes. These sensors are cheaper and require less installation effort than two or even three monoaxial sensors, but they also have the disadvantage of maximum sampling rates in the range of 5000 Hz (e.g., 4500 Hz [25] or 5500 Hz [26]).

For this reason, solutions that can cope with measurements in the frequency range of triaxial accelerometers as inputs are of particular practical interest. To the best of the authors’ knowledge, there are no current solutions that are capable of using only data of sensors with low sampling rates combined with transfer learning between different bearing types. Therefore, a novel transfer-learning-based RUL estimation approach based on data from sensors with low sampling rates for bearings of a different type is presented to fill this research gap. It is based on two feature extraction layers. The first is an intermediate domain-based feature extraction, which focuses on the characteristic fault frequencies of bearings. These frequencies are in a low-frequency range. Because of this intermediate domain, this approach only requires data of a low-frequency range, which can be recorded with low-cost commercial off-the-shelf triaxial accelerometers. The second feature extraction layer is based on convolutional layers, which take the intermediate domain output as the input. The RUL estimation is done with an LSTM-based architecture, which is suitable for transfer learning between different bearing types.

The contributions of this paper can be summarized as follows:

1. It is the first deep transfer learning approach that combines the possibility of using data from low-cost sensors with low sampling frequencies and the usage of transfer learning between different bearing types. This contribution is an important requirement for real-world applications.
2. It is a novel RUL approach that combines a hybrid feature extraction approach (intermediate domain) with a data-driven feature extraction approach (convolutional layers).

This paper is organized as follows: The preliminaries necessary for the presented approach are given in Section 2. Then, the proposed RUL approach is explained in detail in Section 3. This is followed by a validation of the presented RUL approach in Section 4. Finally, the paper concludes with a discussion and an outlook on possible future works in Section 5.

## 2. Preliminaries

### 2.1. Scoring of an RUL Approach

There are many different solutions for classification and RUL tasks [24]. To compare them, a scoring procedure must be used. A particular scoring procedure has been accepted for the RUL estimation of bearings. This scoring procedure was also used during the IEEE PHM 2012 Data Challenge [27], whose challenge was the RUL estimation of bearings. Therefore, it is henceforth called the PHM score in this paper. The dataset as well as the benchmark itself (including the scoring procedure) of this challenge are used in most of the research works for the RUL of bearings [24]. Therefore, this scoring procedure is used without any modifications. As a first step, the relative errors (*Er_i_*) of the predictions have to be calculated according to Equation (1), where *RUL_Act* is the actual RUL and *RUL_Est* is the estimated RUL. The index *i* is for the selected test dataset.

(1) $$Er_{i}=100*\;\frac{RUL\_Act_{i}-RUL\_Est_{i}}{RUL\_Act_{i}}$$

This error rate is used to calculate the weighted error *A_i_* (see Equation (2)). There are two different weights. If *Er_i_* > 0, which means that the estimated RUL is less than the actual RUL, the deviations are less serious than in cases where *Er_i_* < 0. In the first case, a component is replaced too early, resulting only in increased material costs and a short, planned downtime, while the second case leads to an unforeseen and thus unplanned breakdown. Therefore, the two cases are weighted differently.

(2) $$A_{i}=\{\begin{array}{c} e^{-\ln0.5*Er_{i}/5},\;\mathrm{if}\;Er_{i}\leq 0\\ e^{-\ln0.5*Er_{i}/20},\;\mathrm{if}\;Er_{i}>0 \end{array}$$

**Figure 1:**
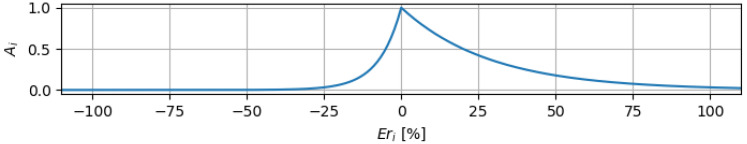
The weighted error Ai (Equation (2)) as a function of the relative error Er_i_. A negative Er_i_ represents a longer estimated RUL than the actual RUL.

The calculated result of this scoring function can be seen in Figure 1.

According to Equation (3), the final PHM score is the mean of the *A_i_*s of all *N* test datasets.

(3) $$score=\frac{\sum_{i=1}^{N}A_{i}}{N}$$

### 2.2. Convolutional Neural Network

Convolutional Neural Networks (CNN) are a specialized type of an artificial neural network. A typical CNN consists of sequential aligned layers. These layers exist of alternating convolutional layers and pooling layers. Finally, a regular feed-forward neural network of fully connected layers is used for the classification task [18].

A convolutional layer exists of neurons that use a small field (window) of the previous layer’s output as the input. All output values inside the window are weighted with specific weights and biases. Then they are combined with the other values in the window and a filter to generate one output pixel.

A pooling layer also uses windows for calculating the output. In contrast to a convolutional layer, a pooling layer aims to reduce the image size by removing unnecessary information/pixels. Therefore, only a simple aggregation function (e.g., max or mean) on the data of the input window is used.

The lower layers of the CNN perform the more generic low-level feature extraction, and the highest layers are used for the classification [28]. The layout of the CNN leads to its ability to maintain information regardless of shift, scale, and distortion invariance [29]. This makes a CNN suitable for analyzing 1D and 2D data, such as time–frequency domain data or classical images.

For a more detailed explanation of CNNs, please refer to the relevant technical literature, e.g., Géron [18].

### 2.3. Long Short-Term Memory Network

An LSTM is a neural network designed to analyze historical time-series data to predict the future [18]. In contrast to a CNN, an LSTM not only uses the current input but also uses data from the previous time steps. The LSTM architecture is based on an inner cell and three gates: input, forget, and output [18]. LSTMs can learn which input is important (input gate) and to what degree it should be stored in the long-term state of the inner cell. With the help of the forget gate, it learns how long to store the input. The output gate is used to set the extent to which the value of the inner cell is forwarded to the next cell. The four elements of an LSTM cell are linked with sigmoid functions and vector and matrix operations. This mechanism is well-suited for long-term patterns such as time series and audio recordings.

From the feature side, an LSTM enhances the classical Recurrent Neural Network (RNN). The gate mechanism is a solution to the ‘vanishing gradient problem’ that an RNN can suffer from [18]. This problem can appear during the backpropagation process of the gradient that is used while training the network. This process starts at the highest layers of the network and uses the gradient to update the neurons’ weights. The gradient can get smaller from layer to layer and finally ‘vanishes’. This results in the lower layers never being trained and the RNN being unable to converge on long-time dependencies.

For a more detailed explanation of LSTMs, please refer to the relevant technical literature, e.g., Emmert-Streib et al. [30].

## 3. Intermediate-Domain-Based RUL Estimation

**Figure 2:**
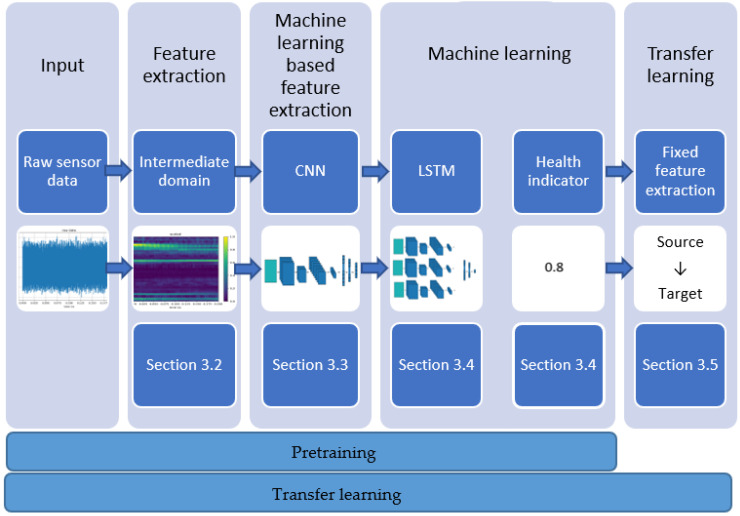
The different stages of the presented transfer learning-based RUL approach. This process is used for the transfer learning step but also for the pretraining of the network.

The starting point for estimating the RUL of bearings is raw sensor data, which comes as time-series data. It can be analyzed in a one-dimensional or two-dimensional way by being converted into the time–frequency domain to obtain the time and the frequency relationship [31]. As described in Section 2.2, CNNs are a suitable approach to analyze 2D data, such as time–frequency domain data or classical images, because of their ability to maintain information regardless of shift, scale, and distortion invariance. In addition to the different measurements over time, which can be analyzed as proposed with a CNN, the time correlations during the degradation process between the different measurements should also be considered. Therefore, input data from previous time steps need to be remembered and used as an additional input for the current time step, which is automatically fulfilled by the working principles of LSTMs (see Section 2.3). Because of this, the proposed and implemented transfer learning RUL approach is based on a CNN for the feature extraction, which is followed by an LSTM for the RUL estimation. This RUL approach is shown in Figure 2 in detail. The feature extraction is based on two layers. The first feature extraction layer is based on an intermediate domain, which prepares the raw sensor data before its usage. In general, the aim of an intermediate domain is to bring the source and the target domain closer together [32]. The proposed intermediate domain, which is a hybrid approach, does this by obtaining advantages from the context information of characteristic frequency bands of the bearings inside the sensor data. These frequency bands are selected by frequency-selective filters. In contrast, other current approaches use pure data-driven approaches for the transformation of the input data (e.g., direct sensor data [33] or wavelet transforms [34]). A benefit of this intermediate domain is that it only requires low-frequency data that can be recorded with today’s triaxial accelerometers. The second feature extraction layer is based on the convolutional layers of a CNN. Both feature extraction techniques are based on our intermediate domain and CNN-based research work, which showed a superior accuracy for the classification of bearing health states to other existing techniques [35]. The high accuracy for the transfer learning tasks is achieved mainly by the intermediate domain, which is used as the input for a CNN.

The extracted features are afterward used as the input of an LSTM, which makes the features time-dependent. The output of this LSTM is a health indicator. A health indicator is a common approach for deep learning based RUL estimation, which transforms timestamps into a more useable abstraction format for neural networks [36,37].

The presented RUL approach can also be used for transfer learning. Therefore, a fixed feature extraction is used. The usage of the intermediate domain brings the source and target domain closer together. This results in a similar and related intermediate domain used for both domains that enhances the transfer learning process.

### 3.1. Feature Extraction: Intermediate Domain

#### 3.1.1. Overview

**Figure 3:**
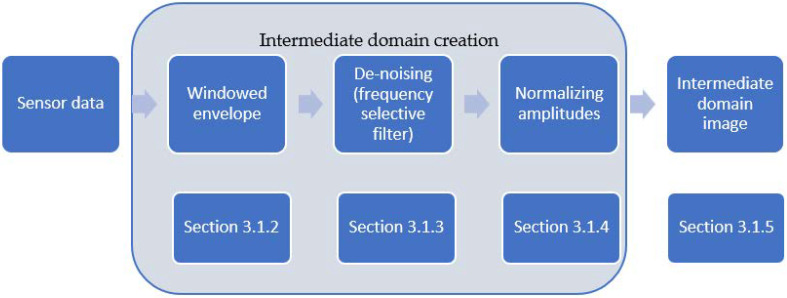
The different steps to obtain the proposed intermediate domain image from the raw sensor data.

An intermediate domain is used to align the features of the input data of the source and target domain to achieve better accuracies in classification and regression models [38]. A common feature of many different mechanical systems is that they consist of components that perform periodic movements. In the case of a bearing, these are the inner ring, the outer ring, the cage, and the balls. In the matter of a defect, each component has its fault pattern based on a mathematically computable fault frequency, with its harmonics and relations to other frequencies. The fault frequencies are used for classical model-based bearing degradation analyses [39]. In this case, they can be extracted from the sensor data with the help of the widely used envelope analysis [40]. In data-driven approaches, the sensor data is used directly [33] or after a transformation into the time–frequency domain [34]. In the latter case, the features are extracted by means of the machine learning algorithm itself. A hybrid approach can close the gap between a model-based approach, which only relies on a potentially imperfect degradation model, and the data-driven approach, which does not consider the physical degradation process [41]. Such a hybrid approach, in the form of an intermediate domain, can optimize the feature extraction, especially in the case of only a small number of training data and different bearing types [35]. Therefore, this section presents an intermediate domain, which is used as the first feature extraction layer of the proposed RUL approach. The presented intermediate domain uses these fault frequencies and converts them to 2D images. As shown in Figure 3, this involves the steps of a windowed envelope, de-noising, and normalizing.

#### 3.1.2. Windowed Envelope

**Figure 4:**
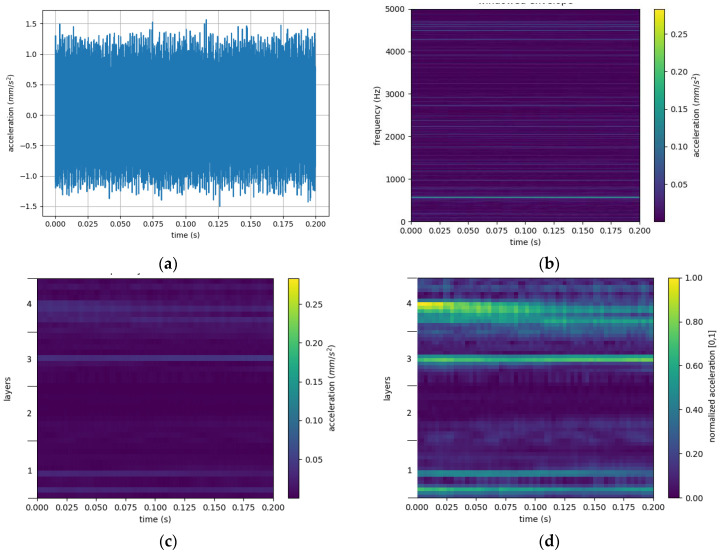
The steps of the intermediate domain creation: (**a**) raw sensor data; (**b**) windowed envelope; (**c**) the frequency selective filter; (**d**) final intermedia domain image (after normalization and scaling). The layers in (**c**,**d**) correspond to the following fault areas of a bearing: 1: outer; 2: inner; 3: ball; 4: cage.

The first step when creating the windowed envelope is to divide the measured values into slices of equal length. Each of the slices is the source of one image. As a next step, the raw sensor data of each slice have to be converted into the time–frequency domain. This is done with the windowed envelope method [35]. This method is comparable to an STFT, in which the time domain data is divided into small segments, and an FFT is performed on each of them. By lining up the FFTs, the frequencies can be displayed as a function of time. For a windowed envelope, the process is similar: A sliding window is used to segment the sensor data in the time domain (see Figure 4a). Afterward, the envelope function is applied to them. This function is based on two steps. First, a Hilbert transform is applied to the input data. This is followed by an FFT to obtain the windowed envelope (see Figure 4b).

#### 3.1.3. De-Noising

The second step in the creation of the intermediate domain is the de-noising of the data based on the specific bearing characteristics. Bearings are made of four relevant components (inner ring, outer ring, cage, and balls), each with specific fault frequencies depending on the current rotational speed and bearing parameters [42]. However, these calculated frequencies are only theoretical values under ideal conditions. They may vary depending on manufacturing tolerances as well as on wear-out conditions of the component. Therefore, frequency bands should be used for the de-noising. Not-yet-published studies related to this work indicate that, in the specific case of bearings, a frequency bandwidth of 10 Hz should be chosen; all data outside the frequency bands around the characteristic component frequencies plus their harmonics can be removed (see Figure 4c). These studies also showed that using four harmonics yields the best results. A big advantage of this method is that, in addition to the de-noising, the error frequencies of each component are always at the same position in the image. This reduces the differences between the source and the target domain for different bearings, which can lead to better results [35].

Using the frequency selective filter, the requirement of using only frequencies of up to 5000 Hz for the commercially available triaxial accelerometers is also fulfilled because these frequencies usually are in the lower four-digit range. Furthermore, specially designed measuring cycles make this even possible for bearings used in high-speed spindles running at up to 23,000 rpm [35].

#### 3.1.4. Normalization

The last step of the intermediate domain creation is the normalization, where the amplitudes of the different frequencies in the image are moved into a range from 0 to 1. The normalization cannot be performed earlier because the now out-filtered other frequencies may have higher amplitudes than the remaining ones and would therefore adulterate the normalization results. The normalization of a specific intermediate domain image (*ID*) is performed by applying Equation (4) to each pixel (ID_P) of the two-dimensional intermediate domain image, where $A_{min}$ is the smallest value of all pixels of all intermediate domain images. Accordingly, $A_{max}$ is the highest value of all pixels among all intermediate domain images. The result is a normalized pixel (*ID_P_Normalized_*).

(4) $$ID\_P_{Normalized}=\frac{ID\_P-A_{min}}{A_{max}-A_{min}}$$

#### 3.1.5. Intermediate Domain Image

Before the image can be used as input of a CNN, the image has to be aligned to the input layer of the CNN. The later-proposed CNN uses a 64 × 64 input size. Therefore, each image is rescaled to a 64 × 64 image: 64 pixels for the timeline and 64 pixels for the 16 areas (4 components and 4 harmonics for the component-specific fault frequencies). This step is undertaken before saving the intermediate domain images to reduce computing time during the training of the neural network later on. The normalized and rescaled image is shown in Figure 4d as the final intermediate domain image.

**Figure 5:**
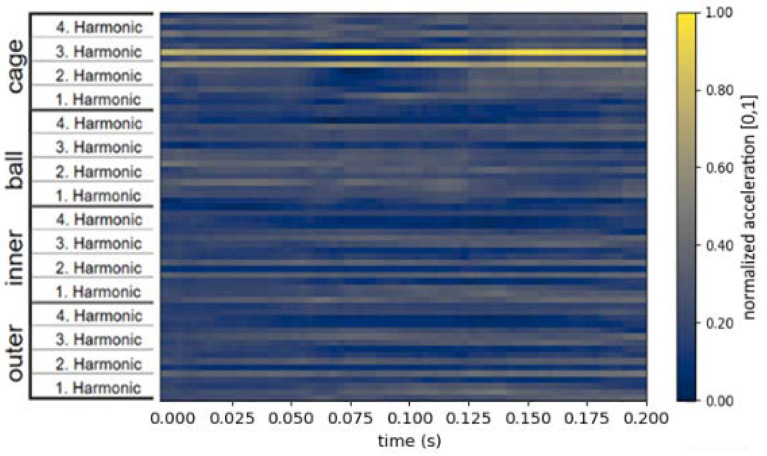
An exemplary intermediate domain image for bearing data with four harmonics for each of the four fault frequencies.

A more detailed example of an intermediate domain image for a bearing is shown in Figure 5. It shows a slice length of 0.2 s and all 16 areas—four harmonics for the characteristic frequencies of the cage, the ball, the inner, and the outer ring.

### 3.2. Feature Extraction: Convolutional Layers

As mentioned previously, the combination of an intermediate domain and a CNN was successfully used in our earlier work for bearing fault classification [35]. The CNN used there is a double-layer CNN. The characteristic of a double-layer CNN is that, instead of a pooling layer after a convolutional layer, a second convolutional layer is used. A pooling layer then follows these two convolutional layers. This layout adds an additional nonlinearity, which increases the significance of the features [31].

This CNN uses fully connected layers for the classification task. These fully connected layers use the output of the convolutional layers. As proposed by Verstraete et al. [31], three fully connected layers have been used for this case, where the last of these layers performs the final classification. In the case of bearings, the last layer has four outputs: one for each of the inner ring, the outer ring, the cage, and the ball fault. In addition, dropout layers with a dropout rate of 0.5 are inserted between the fully connected layers. A dropout layer replaces the value of random neurons according to the dropout rate with zero. As a result, the remaining neurons are trained more independently. This reduces the probability of overfitting because the remaining neurons have to take over the work of the replaced neurons [18].

The complete CNN comprises three consecutive sequences of two convolutional layers and one pooling layer. They are followed by three fully connected layers, where the last fully connected layer is used for the final classification of the bearing state. Between those fully connected layers, a dropout layer is used [35]. This adapts the work of Verstraete et al. [31] and uses a convolutional window of 3 × 3. During the training, cross-entropy, which is a logarithmical loss function, is used.

**Figure 6:**
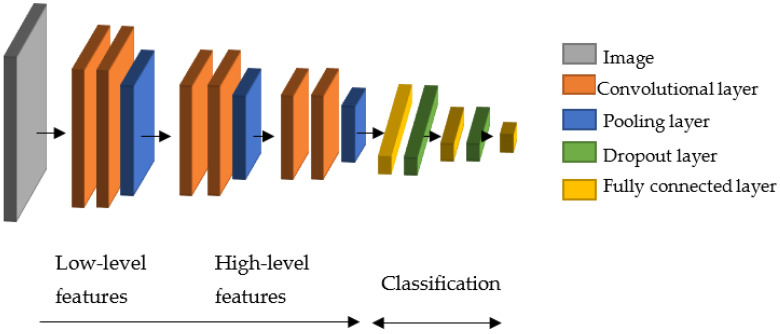
Different tasks of the parts of the presented CNN. They start with low-level feature extraction and end with classification with the help of fully connected layers.

Since the proposed RUL approach uses the same 2D intermediate domain images, the same convolutional layer-based approach can be used for the feature extraction. This second feature extraction mechanism is needed because the RUL approach presented here is based on an LSTM network. Before an LSTM network can use 2D images, the features must be extracted somehow. For this, the feature extraction mechanism of the convolutional layers of a CNN can be used. This results in using the first ten layers of the given CNN for the RUL approach. As shown in Figure 6, the dropout and the fully connected layers are for the classification and are therefore not needed for the RUL task.

### 3.3. Proposed LSTM Architecture

Different deep learning approaches can be used for estimating the RUL, including a CNN, an RNN, and an LSTM. The CNN, however, is unsuitable for establishing a time relationship in a regression problem, as only one input is analyzed at a time, and there is no connection to the previous input values. Higher accuracies can be reached by considering time relations, as is achieved by an RNN or an LSTM [43]. In contrast to CNNs, RNNs and LSTMs also use data from previous time points. As stated in Section 2.3, LSTMs have the advantage of being more robust to the vanishing gradient problem. For these reasons, the presented approach uses an LSTM.

For the layout of the LSTM network, the layout proposed by Sahoo [44] was taken as a starting point because it gives very good results for a sensor-based RUL estimation in the field of turbofan engines. It consists of three layers: the first layer has 128 outputs, the second layer has 64 outputs, and the third layer has 32 outputs. These are followed by fully connected layers. The output of the last fully connected layer, which consists of a single neuron, is used for the RUL estimation based on a health indicator. The used health indicator is in a range between 0 and 1 and has to be transformed back to a time span. This transformation can be performed by means of the linear Equation (5):

(5) $$T_{RUL}=\frac{T_{Cur}}{1-HI}-T_{Cur}$$

where *T_RUL_* is the estimated RUL, *T_CUR_* is the current lifetime of the bearing, and *HI* is the health indicator, which is the output of the RUL network and is in a range between 1.0 for new and 0.0 for a defect [37]. Using a health indicator means that the network is trained with labels between 1.0 and 0.0 instead of a time. Therefore, before the beginning of the training, all training samples must be relabeled.

Based on the above-introduced LSTM, the three modifications listed in the following were evaluated and compared to find a suitable layout for combining an intermediate domain, convolutional layers, and an LSTM. The comparison is based on the PHM score described in Section 2.1, which was selected because of its wide use in the field of the RUL of bearings. The three modifications are explained in the following. They all have the feature extraction part based on the CNN in common.

- Layout 1: This layout reflects an LSTM without any intermediate fully connected layer. It is based only on the feature extraction part of the CNN, followed by the LSTM layout proposed by Sahoo [44], which has 32 outputs after the last LSTM layer. These outputs are directly fed into a fully connected output layer made of one neuron, giving the final health indicator as the output.
- Layout 2: This layout reflects the common usage of several (deep) fully connected layers [45]. Therefore, in addition to Layout 1, another fully connected layer with 32 outputs and a dropout layer with a dropout rate of 0.5 are inserted directly after the last LSTM layer. The use of 32 outputs for the fully connected layer is based on the success of the double convolutional layers in the classification model, where two identical layers are used in a row. Since the previous LSTM layer has 32 outputs, 32 outputs are also chosen here. A dropout factor of 0.5 is chosen based on recommendations in the literature, such as Géron [18].
- Layout 3: This layout only differs from Layout 2 in that the last LSTM layer with 32 outputs is removed. This layout was chosen to analyze the impact of the LSTM layers. A less complex model with fewer layers could be used if this approach was superior.

The three layouts are shown in detail in Table 1. The table also shows the PHM score that can be achieved with each layout. Layout 2 achieved the highest PHM score (0.35) and was therefore chosen for the final RUL framework.

**Figure 7:**
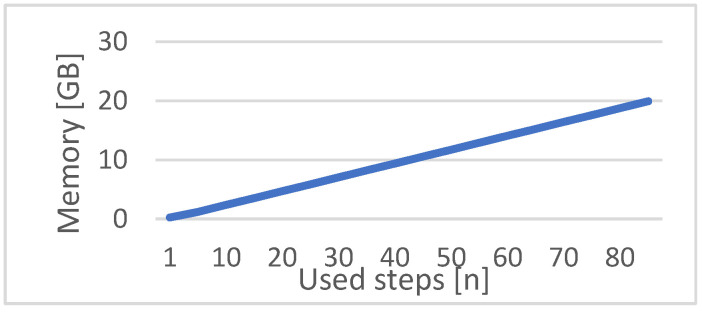
Theoretically needed memory for training with different numbers of used steps.

**Figure 8:**
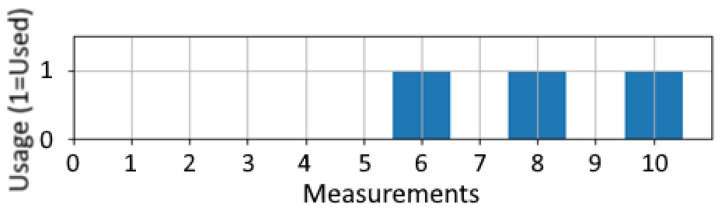
Example of the window size with 10 measured values. The step size *s* = 2 and the number of used steps *n* = 3.

Another important factor is the window size of the LSTM input. A bigger window size means that measurements of more previous time steps are considered in the calculation. However, this advantage comes at the price of two disadvantages. The first is that a minimum length of the selected window size must always be available for testing as well as for training. Therefore, short-time intervals cannot be analyzed. The second disadvantage is the required memory, especially during the training of the network. As shown in Figure 7, the memory increases depending on the window size, which is especially noticeable when using images as input, like the intermediate domain. It is only a linear growth, but the required memory quickly exceeds 16 GB, which is the maximum memory of the current mainstream graphic cards used in commercial clouds, e.g., an NVIDIA P100 [46]. The window size depends on two factors: First, the total number of steps *n*, which defines how many measurements should be used. This size is equal to the neural network’s input layer. The second factor is the step size *s*, which specifies the distance between the used measurements. The multiplication of *n* and *s* calculates the covered window of measured values. Figure 8 shows an example with a total measurement range of 10. By setting the parameters *s* to 2 and *n* to 3, the measurements 6, 8, and 10 are used as input for the RUL estimation at measurement point 10.

**Figure 9:**
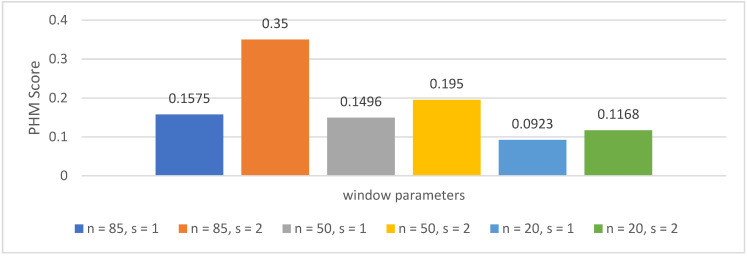
Evaluation of different numbers of used steps (*n*) and step sizes (*s*) for the training of the proposed RUL framework. The best result is achieved with an input size of 85 and a step size of 2.

An evaluation was conducted to determine a suitable combination of *n* and *s*. The results, which can be seen in Figure 9, indicate that the larger the total window size, the better the PHM score. In the example of the datasets of the PHM Data Challenge, the maximum length based on the smallest test dataset is 171 measurements. As described above, the number of usable measurements is limited by the hardware used. This limit is also true for the given case where not all measurements can be used together, which leads to the largest possible window size of 170 (*n* = 85, *s* = 2). The evaluation also reveals that, by using the same number of steps *n*, a higher step size *s* leads to an increased PHM score. Based on this evaluation, a step size of 2 and the usage of 85 steps are suggested for the given setup. For other datasets (e.g., with larger possible window sizes), the evaluation has to be conducted again.

### 3.4. Transfer Learning Approach

The presented RUL approach is fully functional even without transfer learning. As mentioned previously, a widespread problem with predictive maintenance solutions is the lack of datasets. Therefore, an approach that can benefit from datasets of a different domain has significant importance for real-life scenarios where only a few samples are available. The presented approach is well suited for transfer learning since it is based on the intermediate domain and the convolutional layers for the feature extraction. The intermediate domain creates similar images for different bearing types. The convolutional layers can benefit from the similarity of the images, as it leads to a similar or nearly the same feature extraction mechanism.

A common transfer learning type is the fixed-feature extraction [28]. As mentioned in Section 2.2, deep learning models have in common that the first layers are for low-level feature extraction and the latter for high-level feature extraction. In a fixed-feature extraction, the model is pretrained with a source domain dataset. Afterward, all layers that are used only for the feature extraction are fixed. For instance, in the case of a CNN, the feature extraction is performed in the convolutional layers. Therefore, a fixed-feature extraction of a CNN is a transfer learning approach where the weights of the convolutional layers are fixed. Only the fully connected layers that are used for the classification are retrained with the target dataset. Since the intermediate domain creates very similar input data, the feature extraction does not change much between the two domains. Therefore, the fixed-feature extraction approach should fit very well. Only the usage of the extracted features may be different, so the RUL estimation part has to be adapted.

There is another advantage in using the method of fixed-feature extraction for the convolutional layers, especially for bearings. It is often the case that there are extensive datasets available for classification but only small ones for the RUL estimation. With this approach, however, the CNN for classification tasks, as described in Section 3.2, can be trained with a classification dataset. Afterward, the weights of the trained convolutional layers can be transferred to the convolutional layers of the RUL approach. Two separate training runs of the IEEE PHM 2012 Data Challenge datasets were performed to validate this. One of the runs used pretrained convolutional layers. These layers were trained with the drive-end bearing datasets of Case Western Reserve University [23].

**Figure 10:**
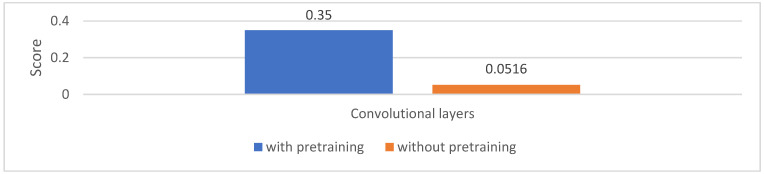
The scores for the RUL estimation with and without pretraining of the convolutional layers. The score achieved with pretrained layers is much higher than that without pretraining.

The detailed results in terms of the relative errors *Er* of both runs are shown in Table 2. For 10 out of 11 bearings, the run with pretraining is better than the one without it, and the mean *Er* is reduced to 707 vs. 1213. With the help of these *Er*s, the final PHM score for the RUL estimation can be calculated (see Section 2.1). As can be seen in Figure 10, the score with pretrained convolutional layers is much higher than that without pretraining. Another benefit of using transfer learning, as mentioned before, is the saving of computing resources. The memory needed for training convolutional layers is around three times the memory required for using trained convolutional layers [47]. Using this knowledge combined with the findings of Figure 7, the approximate memory consumption expected for each *n* can be determined.

All tests in Section 3.3 were also performed with this pretrained dataset to obtain better results and to obtain a suitable solution for the studied task of transfer learning between different bearing types.

### 3.5. Constraint

**Figure 11:**
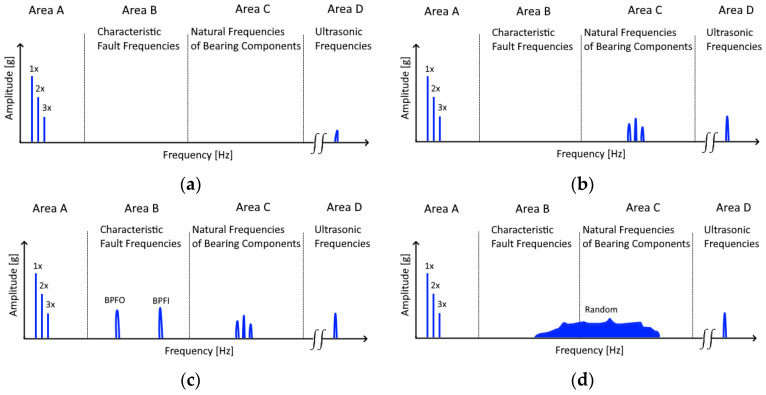
The four bearing degradation stages: (**a**) stage 1, contains the rotation frequencies and ultrasonic frequencies; (**b**) stage 2, the natural frequencies of the bearing become visible; (**c**) stage 3, the characteristic fault frequencies appear; (**d**) stage 4, the frequencies in area B and area C are replaced with random noise.

The proposed approach also has a constraint that is based on the used intermediate domain. This constraint becomes apparent by looking at the four degradation stages of a bearing [48,49]. These stages are also shown in Figure 11. The first stage, in which a crack is just developing, is only visible in the ultrasonic frequency range. In stage 2, the crack increases, which creates impact forces sufficient to excite the natural frequencies of the various bearing components [50]. These frequencies cannot be determined by simple formulas and must be determined empirically. They are usually in the range of 2–6 kHz [49]. In the third stage, the bearing degradation continues, and small parts of the defective bearing component may come off. This causes the characteristic fault frequencies to appear. It is the first stage that can be used for a reliable analysis. Therefore, current condition monitoring and predictive maintenance systems primarily focus on this stage [40]. In the last stage, before the total failure, some severe flaws may be fixed with metal parts removed from other flaws and then smoothed over by the rolling elements. In addition, the clearance within the bearing increases. As a result, the intensity of the previously visible characteristic fault frequencies may decrease again. Instead, random frequencies appear in the form of background noise.

Since the proposed intermediate domain relies on the characteristic fault frequencies that appear in the degradation stage 3, it cannot detect incipient bearing damage in the fault in stage 2. However, this is not only a problem of the used intermediate domain but also of the triaxial sensors, which are the focus of this research work. These sensors, which have a maximum resolution of 5000 Hz, can also not detect every natural frequency of the bearing components. Nevertheless, this is not a big problem for a real-life scenario since, in stage 3, 1% to 5% of the expected life of the bearing still remains [49]. Therefore, this is still enough time for planned maintenance.

### 3.6. Generalization

The proposed transfer learning approach consists of three parts: intermediate domain, convolutional layers, and LSTM layers. In order to provide a general solution for different bearing types, it is essential to know which parts have to be adapted and which can be directly transferred.

The intermediate domain is dependent only on the physical parameters of the used bearing. Based on these parameters, the characteristic fault frequencies are calculated. All other parameters can be kept constant. This was verified with the Case Western Reserve University dataset for the RUL estimation of the IEEE PHM 2012 Data Challenge dataset (see Section 3.4).

The architecture of the convolutional layers can be used for all kinds of bearings without modification because the input is always the intermediate domain with the same characteristics. This assumption can also be taken over to the LSTM architecture. However, one exception is the window size (see Section 3.3). The window size must be adjusted depending on the dataset and available hardware.

## 4. Benchmark

To validate the presented RUL approach, a benchmark was performed. For this purpose, the IEEE PHM 2012 Data Challenge dataset was chosen due to its widespread use and good documentation. This also includes the benchmark setup and scoring procedures. Hosted by the IEEE Reliability Society and the FEMTO-ST Institute, this challenge took place in 2012. To the best of the authors’ knowledge and additional sources such as [5], there is still no more recent or better reference dataset available. Unfortunately, as described in Section 1, the works of Xia et al. [21] and of Huang et al. [13], which are the only other current transfer learning approaches between different bearing types, do not use this benchmark. Instead, they use the IEEE PHM 2012 Data Challenge dataset in combination with a custom benchmark that is not described in detail. Therefore, our presented RUL approach cannot be compared to their approaches.

This section is divided into four parts. The first describes the benchmark. This is followed by supplements to the test positions. Afterward, the execution of the benchmark itself is provided. Lastly, a conclusion summarizes the results.

### 4.1. Benchmark Description

The IEEE PHM 2012 Data Challenge focused on estimating the RUL of bearings and was open to both industrial and academic participants. All participants had access to datasets from 17 different test runs provided by FEMTO-ST. Each test run was recorded with a horizontal and a vertical accelerometer. Each test run was terminated as soon as an acceleration of more than 20 g was reached. The accelerometer had a sampling frequency of 25.6 kHz. Therefore, other approaches that use data from the whole available frequency range might have better results than the proposed approach, which focuses on data from sensors with low sampling rates. The datasets, available at three different operating conditions, were split into six learning and 11 test datasets (see Table 3). The test datasets were truncated to a random length to estimate the RUL. The winners were chosen based on the PHM score presented in Section 2.1.

### 4.2. Supplements to the Test Positions

The test position of the test dataset can be in different degradation stages, such as stage 2 or 3. The IEEE PHM 2012 Data Challenge has two datasets in stage 2. To illustrate this, a dataset in stage 3 (bearing 1_4) and the two datasets in stage 2 (bearing 1_6 and bearing 2_5) are analyzed in the following. Therefore, two plots are used for each dataset: a time domain plot that shows the amplitudes of the horizontal and vertical accelerometer values. In addition, the test position is marked, and a time–frequency plot in the frequency range of the fault frequencies of the degradation stage 3 is shown. Here, the amplitudes of the horizontal accelerometers are shown based on an FFT calculated every 500 s.

#### 4.2.1. Bearing Dataset 1_4

**Figure 12:**
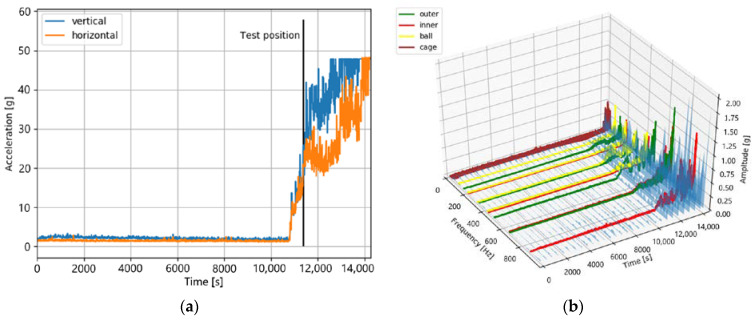
The sensor values of bearing dataset 1_4 in the time domain (**a**) and in the time–frequency domain (**b**). Both figures show increased amplitudes at the test position.

This dataset has a total length 14,280 s. A lifetime of 11,390 s was defined as the test position. According to Figure 12, this dataset is in degradation stage 3.

This assumption is based on the time domain plot in Figure 12a that shows that, at the test position, both accelerometers have an increased acceleration value. These acceleration values show an increasing trend until the end of the lifetime. This characteristic also matches the characteristics in the time–frequency domain, shown in Figure 12b, which shows increased amplitudes at the test position, especially at the fault frequencies of degradation in stage 3.

#### 4.2.2. Bearing Dataset 2_5

**Figure 13:**
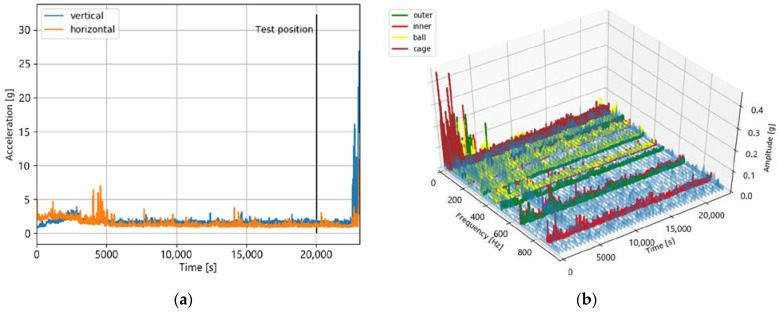
The sensor values of bearing dataset 2_5 in the time domain (**a**) and the time–frequency domain (**b**). Both plots do not indicate a degradation at the test position.

This dataset has a total length of 23,110 s. The test position is after a lifetime of 20,020 s. As can be seen in Figure 13, there are no indications of a degradation of stage 3. This degradation stage starts only after approximately 23,000 s of lifetime.

#### 4.2.3. Bearing Dataset 1_6

This is the second dataset which is probably in degradation stage 2. It has a total length of 24,480 s, and the test position is after a lifetime of 23,020 s.

**Figure 14:**
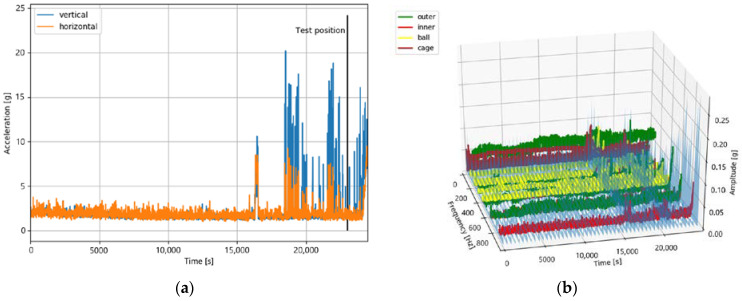
The sensor values of bearing dataset 1_6 in the time domain (**a**) and in the time–frequency domain (**b**). Both plots show no increased measured values at the test position after 23,020 s.

Figure 14 shows that, as well as in the case of bearing 2_5, there are no indications of a monotonous degradation either in the time domain or in the time–frequency domain at the test position. The degradation of stage 3 starts at approximately 24,000 s. It is worth mentioning that the measurement has some short-term high peaks before the test position of an unknown source. Furthermore, compared to bearing 1_4 and bearing 2_5, there is a strong scattering of the measured values over the entire bearing lifetime.

### 4.3. Benchmark Execution

This benchmark was executed under the same conditions as in the IEEE PHM 2012 Data Challenge. In addition, the drive-end dataset of Case Western Reserve University [23] was used for the proposed transfer learning approach, as described in Section 3.4. Afterward, the pretrained RUL network was trained with the following parameters:

- Used environment: The relevant components are an NVIDIA P100 GPU in combination with Python (version 3.6.8) and the TensorFlow machine learning library (version 2.2).
- Intermediate domain: The used bearing, in combination with the used process parameters, results in the following characteristic fault frequencies: outer ring fault: 168 Hz, inner ring fault: 222 Hz, ball fault: 108 Hz, and cage fault: 13 Hz. This results in a maximum frequency of 888 Hz, which is required for the fourth harmonics of the inner ring fault. The intermediate domain thus fulfills the requirement of having a solution that can be used in use cases with current industrial triaxial accelerometers with low sampling rates.
- LSTM layout: The intermediate domain images were used in an LSTM, according to Section 3.3. Among other parameters, a window size based on 85 measurements (*n* = 85) and a step size of two (*s* = 2) were used.
- Training settings: A batch size of 120 was used for the training. A larger batch size could not be used because of hardware limitations. In addition, a learning rate of 0.0005 was used. An Adam optimizer with the mean squared error (MSE) as a loss function was used during the training. This was based on the recommendation of Liu et al. [12] that, of all common loss functions, MSE is the most sensitive to measurement errors. For the training itself, 300 epochs were used since no improvements in the result of the loss function could be achieved afterward.

Subsequently, the trained network was tested with the test datasets. The results in the form of the relative error (*Er*), its mean, and the PHM score are presented in Table 4. In addition, the results of Sturisno et al. [51] (winner of the academics), Porotsky and Bluvband [52] (winner of the industrial), Zheng [53] (a current work), and Zhang et al. [54] are also presented. The approach of Zhang et al. is the best current one in terms of PHM score and mean relative error. In addition, they compared their PHM score to those of other current approaches, which have PHM scores in a range between 0.26 and 0.62. All the above-presented works used pure data-driven approaches, which do not rely on any physical parameters.

Using only the result of the PHM score, which was the relevant metric for the IEEE PHM 2012 Data Challenge, as a metric, the presented approach is superior to the two winning approaches of the challenge and many others, such as Zheng [53]. Unfortunately, the presented approach is the worst when looking at the relative error. Caused by two outliers, which have a relative error of −6413.71% (bearing 1_6) and −919.58% (bearing 2_5), the mean relative error is 707.42%. These datasets are of bearings in degradation stage 2. If the benchmark is conducted without these two datasets, the mean relative error decreases to 40.76%, which is again a good result. It is noteworthy that, for the case of Sturisno et al., the highest deviation is also for bearing dataset 2_5.

Both outliers of the presented approach (bearing 1_6 and bearing 2_5) have a negative value of *Er*. According to Equation (3), a negative *Er* represents a too-large estimated RUL. This behavior is based on the constraint mentioned in Section 3.5 for this approach: the bearing has to be at degradation stage 3, at least, to emit the fault frequencies that are used by the intermediate domain. If these frequencies have no increased amplitude yet, the proposed approach cannot detect a degradation leading to the too-large estimated RUL.

There are also current approaches, such as the one by Zhang et al. [54], that are superior to the presented one. To the best of the authors’ knowledge, all superior approaches use features of the time–frequency domain as the input. In contrast to the presented approach, which uses only frequencies less than 900 Hz, these approaches use the complete possible frequency range of the datasets, which is up to 12,800 Hz. This results in having more features for the RUL estimation, which enhances the results.

### 4.4. Conclusion

The performed benchmark showed that very good results can be achieved by the presented RUL approach in combination with transfer learning by using only low-frequency features. Even a dataset of an entirely different bearing can be used for transfer learning. The results are even better than the winning approaches of the IEEE PHM 2012 Data Challenge. This is especially remarkable since the presented approach does not use frequencies above 900 Hz. For most of the used test datasets, the estimated RUL is close to the actual RUL. Two datasets were not estimated correctly. This is because this approach, which is based on the intermediate domain created by the characteristic fault frequencies, is optimized for the RUL estimation inside the degradation area of the characteristic fault frequencies. The test positions of the two outlier datasets are before the beginning of this degradation area. Therefore, for a real-world scenario using this approach, the RUL estimation should be started only if a degradation is already recognizable, because only then a realistic RUL value can be determined.

The few other current approaches that reach a higher PHM score, such as the one of Zhang et al. [54], use the time–frequency domain. They consider the natural frequencies of the bearing components Hz, 6000 Hz, and 12,000 Hz, for their RUL estimation by using the whole bandwidth of the available frequencies. In fact, some approaches even focus on these high-frequency ranges, for example, the work of Yoo and Baek [55], which is the second-best current approach with a PHM score of 0.62. Instead, the proposed intermediate-domain-based approach does have a smaller frequency range for the analysis, which leads to worse preconditions and therefore lower performance in contrast to some other approaches. However, although the intermediate domain does not cover the natural frequencies (degradation stages 2 and 3), these frequencies also cannot be recorded with the currently available industrial triaxial sensors. These sensors, which are the focus of this work, often have a maximal sampling rate of about 5000 Hz (see Section 1). Therefore, as described in Section 3.5, this is also a limitation of the usage of the targeted sensors. Due to this limitation, the proposed approach can only detect defects in degradation stage 3. For this reason, we believe it can be justified to skip the two datasets from stage 2 (bearing 1_6 and bearing 2_5).

Although the used benchmark contains test positions at degradation stage 2, this benchmark is used because it is the most widespread benchmark for the RUL estimation of bearings. In addition, no publicly available dataset contains only data with low sampling frequencies.

## 5. Discussion and Future Work

This research work presented a new approach for estimating the RUL of bearings for accelerometers with low sampling rates based on an LSTM and intermediate-domain-based transfer learning. This framework can increase the accuracy for small datasets through transfer learning of knowledge of a different bearing type. These datasets do not even have to be RUL datasets; it is also possible to use datasets that are for the bearing classification. This significantly increases the usability of the transfer learning approach because other well-documented, publicly available datasets can be used. In addition, the presented approach can be used with today’s triaxial accelerometers, which often have sampling rates in the range of 5000 Hz. Using these accelerometers decreases the costs of material and the wiring complexity. The process was verified with a benchmark based on the IEEE PHM 2012 Data Challenge. This benchmark demonstrates the effectiveness of the presented approach based on the PHM score. It reached an even higher score than the winning approaches of the IEEE PHM 2012 Data Challenge. In addition, the above-mentioned possibility of using a classification dataset of a different bearing for transfer learning was proven with a classification dataset of Case Western Reserve University. Furthermore, the capability of using triaxial sensors was also proven since the intermediate domain for this use case only used frequencies of up to 900 Hz.

At the same time, the constraint of this approach became apparent through the two test positions in degradation stage 2. This constraint is that the intermediate domain is unsuitable for cases where the degradation based on the characteristic fault frequencies has not started yet. This is the case when the bearing is in the second degradation stage, where only excited natural frequencies of the bearing components appear. For an industrial use case, this constraint is not of significant consequence because, at the time point of the beginning of such a degradation, there is still enough time for planning a maintenance service. Starting with the third degradation stage, which is based on the fault frequencies, this approach delivers accurate RUL times, which is important for the industrial use case. The few other approaches that reach higher PHM scores consider the frequency ranges of the exciting natural frequencies or even focus on them. Accordingly, they achieve better results than the presented approach. However, they do not fulfill the requirement that they can be used for industrial triaxial sensors.

There are three suggested directions for future research: first, validate this approach with other bearing datasets; second, adopt this approach to other components with characteristic fault frequencies, such as gears; third, improve the RUL estimation for measurements in the lower frequency ranges.

## Figures and Tables

**Table 1:** The three evaluated layouts for the RUL approach. The number of outputs of each layer is in parentheses. The evaluation was conducted with the IEEE PHM 2012 Data Challenge dataset. Except for the differences in the table, all three evaluations were conducted with the same settings.

	Layout 1	Layout 2	Layout 3
Used Layers	CNN (8192)	CNN (8192)	CNN (8192)
	LSTM (128)	LSTM (128)	LSTM (128)
	LSTM (64)	LSTM (64)	LSTM (64)
	LSTM (32)	LSTM (32)	Fully Connected (32)
	Fully Connected (1)	Fully Connected (32)	Dropout (rate = 0.5)
		Dropout (rate = 0.5)	Fully Connected (1)
		Fully Connected (1)	
PHM Score	0.1094	0.35	0.05647

**Table 2:** Detailed results of the proposed RUL framework with and without transfer learning. For each of the 11 bearings, the relative error *Er* according to Equation (1) is shown. In addition, the mean of all values is given.

Bearing	Without Pretraining [*Er*]	With Pretraining [*Er*]
1_3	−70.56	29.27
1_4	−172.46	−78.35
1_5	−968.26	−159.24
1_6	−10,570.0	−6413.71
1_7	−268.18	35.37
2_3	16.34	−0.7
2_4	−323.12	−124.18
2_5	−384.43	−919.58
2_6	−251.93	8.16
2_7	−86.63	12.13
3_3	−234.16	−0.96
Mean	1213.28	707.42

**Table 3:** Assignment of the different datasets to test and training data.

Datasets	Operating Conditions		
	1800 rpm; 4000 N Load	1650 rpm; 4200 N Load	1500 rpm; 5000 N Load
Learning set	Bearing1_1	Bearing2_1	Bearing3_1
	Bearing1_2	Bearing2_2	Bearing3_2
Test set	Bearing1_3	Bearing2_3	Bearing3_3
	Bearing1_4	Bearing2_4	
	Bearing1_5	Bearing2_5	
	Bearing1_6	Bearing2_6	
	Bearing1_7	Bearing2_7	

**Table 4:** This table shows the relative error *(Er)*, its mean, and the PHM score of the different RUL approaches and the proposed approach (with and without bearing datasets 1_6 and 2_5).

Bearing	Sutrisno et al. [51] (%)	Porotsky and Bluvband [52] (%)	Zheng [53] (%)	Zhang et al. [54](%)	Proposed RUL Framework (%)	Proposed RUL Framework without 1_6 and 2_5 (%)
Bearing 1_3	97	N/A	92.44	2.27	29.27	29.27
Bearing 1_4	80	N/A	100	5.6	−78.35	−78.35
Bearing 1_5	9	N/A	20.43	12.42	−159.24	−159.24
Bearing 1_6	−5	N/A	7.76	10.96	−6413.71	N/A
Bearing 1_7	−2	N/A	82.29	−22.46	35.37	35.37
Bearing 2_3	64	N/A	82.93	0.99	−0.7	−0.7
Bearing 2_4	10	N/A	3.22	5.76	−124.18	−124.18
Bearing 2_5	−440	N/A	58.77	25.89	−919.58	N/A
Bearing 2_6	49	N/A	5.63	−10.85	8.16	8.16
Bearing 2_7	−317	N/A	−121.94	1.72	12.13	12.13
Bearing 3_3	90	N/A	−54.38	−3.66	−0.96	−0.96
Mean	105.73	N/A	57.25	9.32	707.42	40.76
Score	0.3066	0.28	0.2992	0.64	0.35	0.43

## Data Availability

This article used two publicly available datasets: 1. Bearing datasets of the Case Western Reserve University: https://engineering.case.edu/bearingdatacenter/download-data-file (accessed on 1 July 2022); 2. Bearing dataset of FEMTO-ST used at the IEEE PHM 2012 Data Challenge: There is no official download source anymore.
